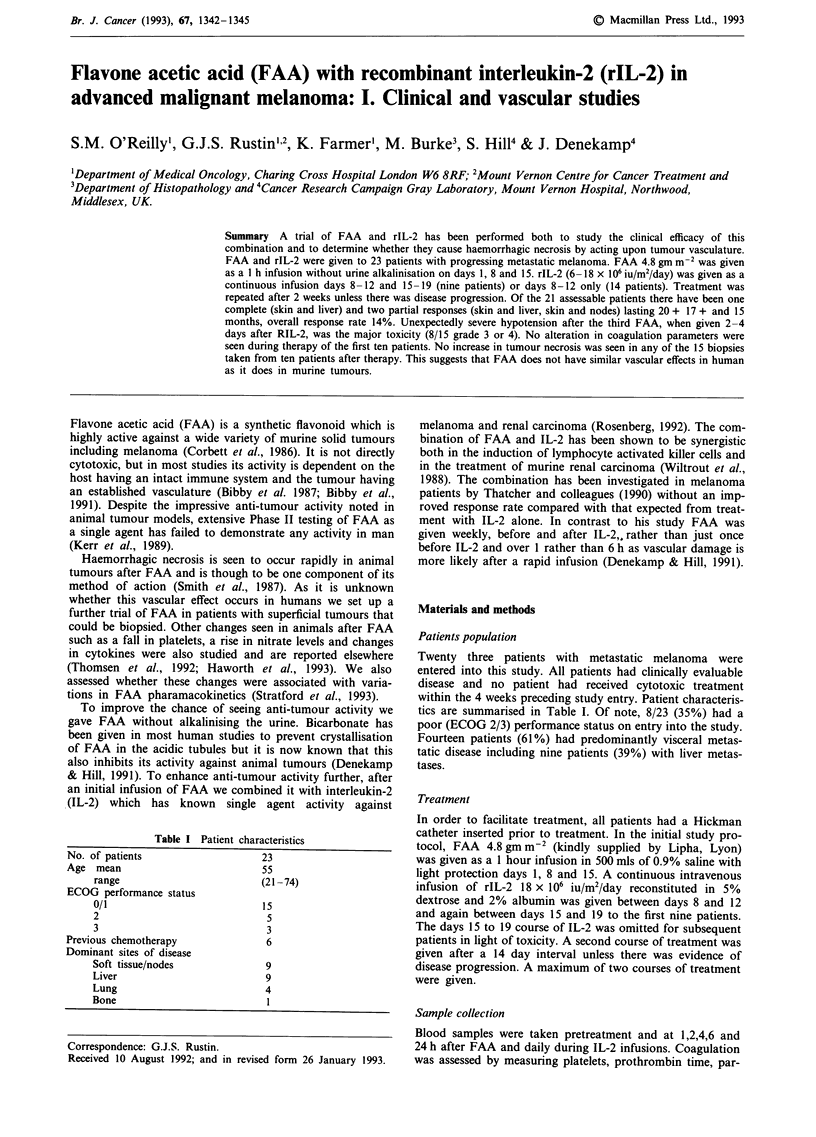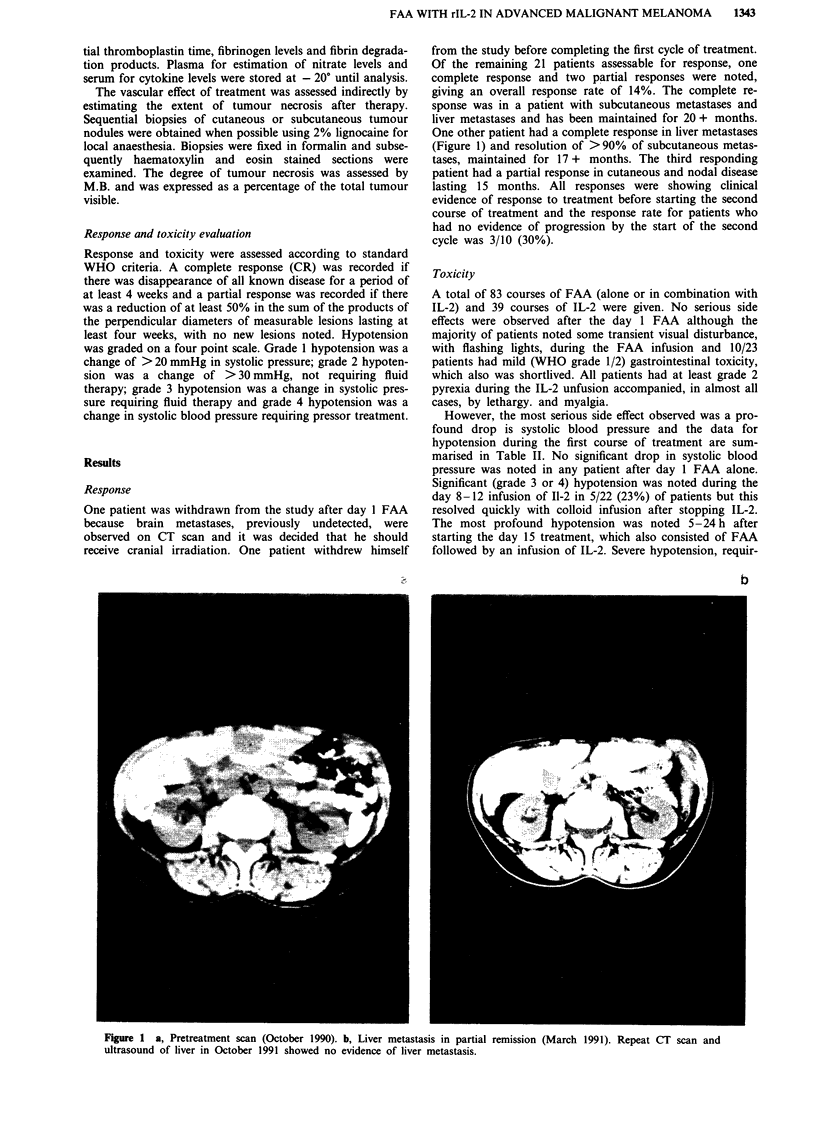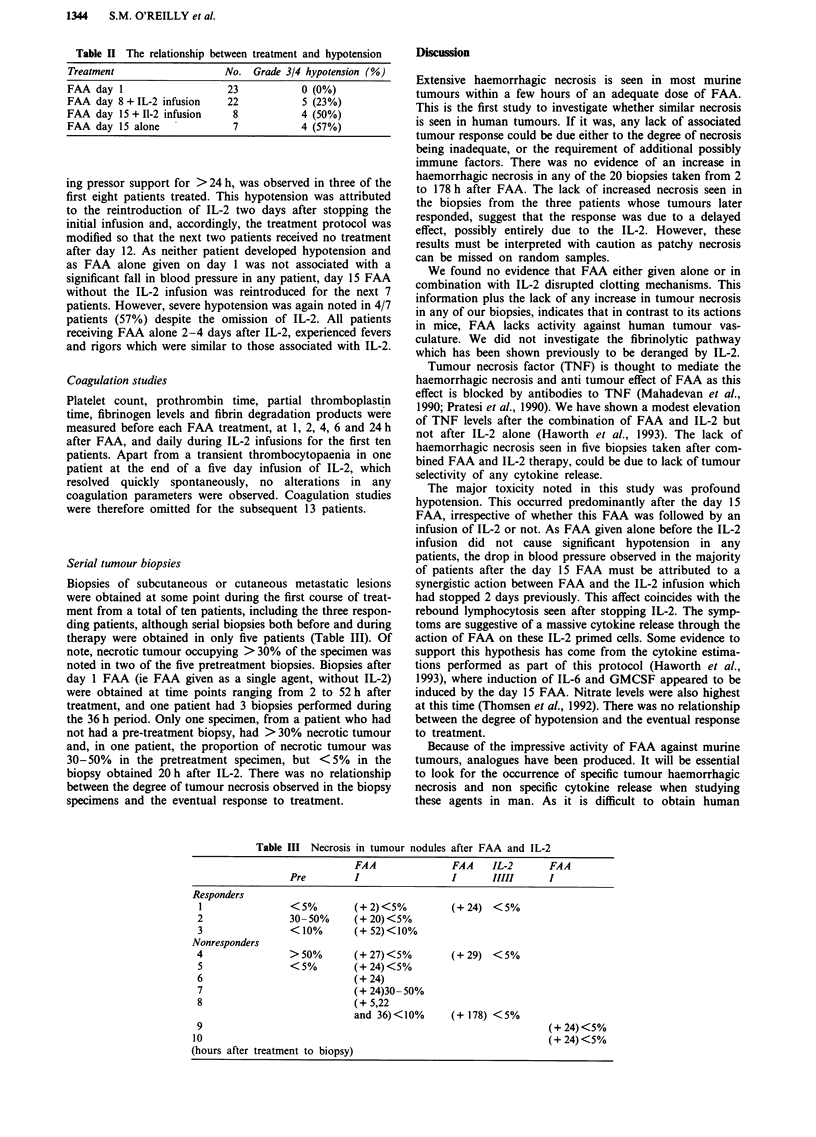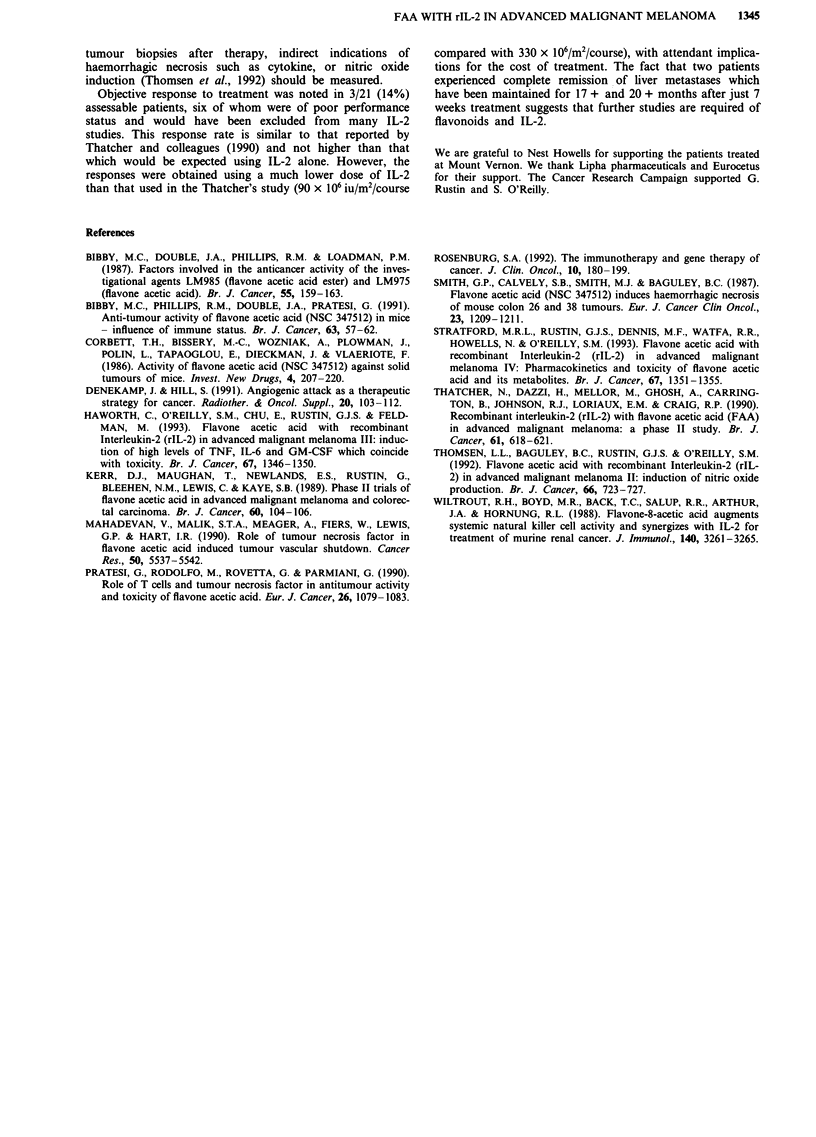# Flavone acetic acid (FAA) with recombinant interleukin-2 (rIL-2) in advanced malignant melanoma: I. Clinical and vascular studies.

**DOI:** 10.1038/bjc.1993.248

**Published:** 1993-06

**Authors:** S. M. O'Reilly, G. J. Rustin, K. Farmer, M. Burke, S. Hill, J. Denekamp

**Affiliations:** Department of Medical Oncology, Charing Cross Hospital London, UK.

## Abstract

**Images:**


Br. J. Cancer (1993), 67, 1342–1345 © Macmillan Press Ltd., 1993

### Flavone acetic acid (FAA) with recombinant interleukin-2 (rIL-2) in advanced malignant melanoma: I. Clinical and vascular studies

S.M. O'Reilly[1], G.J.S. Rustin[1,2], K. Farmer[1], M. Burke[3], S. Hill[4] & J. Denekamp[4]

[1]*Department of Medical Oncology, Charing Cross Hospital London W6 8RF;* [2]*Mount Vernon Centre for Cancer Treatment and* [3]*Department of Histopathology and* [4]*Cancer Research Campaign Gray Laboratory, Mount Vernon Hospital, Northwood, Middlesex, UK.*

**Summary** A trial of FAA and rIL-2 has been performed both to study the clinical efficacy of this combination and to determine whether they cause haemorrhagic necrosis by acting upon tumour vasculature. FAA and rIL-2 were given to 23 patients with progressing metastatic melanoma. FAA 4.8 gm $m^{-2}$ was given as a 1 h infusion without urine alkalinisation on days 1, 8 and 15. rIL-2 (6–18 × $10^6$ iu/$m^2$/day) was given as a continuous infusion days 8–12 and 15–19 (nine patients) or days 8–12 only (14 patients). Treatment was repeated after 2 weeks unless there was disease progression. Of the 21 assessable patients there have been one complete (skin and liver) and two partial responses (skin and liver, skin and nodes) lasting 20 + 17 + and 15 months, overall response rate 14%. Unexpectedly severe hypotension after the third FAA, when given 2–4 days after RIL-2, was the major toxicity (8/15 grade 3 or 4). No alteration in coagulation parameters were seen during therapy of the first ten patients. No increase in tumour necrosis was seen in any of the 15 biopsies taken from ten patients after therapy. This suggests that FAA does not have similar vascular effects in human as it does in murine tumours.

Flavone acetic acid (FAA) is a synthetic flavonoid which is highly active against a wide variety of murine solid tumours including melanoma (Corbett *et al.*, 1986). It is not directly cytotoxic, but in most studies its activity is dependent on the host having an intact immune system and the tumour having an established vasculature (Bibby *et al.* 1987; Bibby *et al.*, 1991). Despite the impressive anti-tumour activity noted in animal tumour models, extensive Phase II testing of FAA as a single agent has failed to demonstrate any activity in man (Kerr *et al.*, 1989).

Haemorrhagic necrosis is seen to occur rapidly in animal tumours after FAA and is though to be one component of its method of action (Smith *et al.*, 1987). As it is unknown whether this vascular effect occurs in humans we set up a further trial of FAA in patients with superficial tumours that could be biopsied. Other changes seen in animals after FAA such as a fall in platelets, a rise in nitrate levels and changes in cytokines were also studied and are reported elsewhere (Thomsen *et al.*, 1992; Haworth *et al.*, 1993). We also assessed whether these changes were associated with variations in FAA pharamacokinetics (Stratford *et al.*, 1993).

To improve the chance of seeing anti-tumour activity we gave FAA without alkalinising the urine. Bicarbonate has been given in most human studies to prevent crystallisation of FAA in the acidic tubules but it is now known that this also inhibits its activity against animal tumours (Denekamp & Hill, 1991). To enhance anti-tumour activity further, after an initial infusion of FAA we combined it with interleukin-2 (IL-2) which has known single agent activity against melanoma and renal carcinoma (Rosenberg, 1992). The combination of FAA and IL-2 has been shown to be synergistic both in the induction of lymphocyte activated killer cells and in the treatment of murine renal carcinoma (Wiltrout *et al.*, 1988). The combination has been investigated in melanoma patients by Thatcher and colleagues (1990) without an improved response rate compared with that expected from treatment with IL-2 alone. In contrast to his study FAA was given weekly, before and after IL-2,, rather than just once before IL-2 and over 1 rather than 6 h as vascular damage is more likely after a rapid infusion (Denekamp & Hill, 1991).

**Table I** Patient characteristics

| | |
|---|---|
| No. of patients | 23 |
| Age mean | 55 |
| range | (21–74) |
| ECOG performance status | |
| 0/1 | 15 |
| 2 | 5 |
| 3 | 3 |
| Previous chemotherapy | 6 |
| Dominant sites of disease | |
| Soft tissue/nodes | 9 |
| Liver | 9 |
| Lung | 4 |
| Bone | 1 |

### Materials and methods

### *Patients population*

Twenty three patients with metastatic melanoma were entered into this study. All patients had clinically evaluable disease and no patient had received cytotoxic treatment within the 4 weeks preceding study entry. Patient characteristics are summarised in Table I. Of note, 8/23 (35%) had a poor (ECOG 2/3) performance status on entry into the study. Fourteen patients (61%) had predominantly visceral metastatic disease including nine patients (39%) with liver metastases.

### *Treatment*

In order to facilitate treatment, all patients had a Hickman catheter inserted prior to treatment. In the initial study protocol, FAA 4.8 gm $m^{-2}$ (kindly supplied by Lipha, Lyon) was given as a 1 hour infusion in 500 mls of 0.9% saline with light protection days 1, 8 and 15. A continuous intravenous infusion of rIL-2 18 × $10^6$ iu/$m^2$/day reconstituted in 5% dextrose and 2% albumin was given between days 8 and 12 and again between days 15 and 19 to the first nine patients. The days 15 to 19 course of IL-2 was omitted for subsequent patients in light of toxicity. A second course of treatment was given after a 14 day interval unless there was evidence of disease progression. A maximum of two courses of treatment were given.

### *Sample collection*

Blood samples were taken pretreatment and at 1,2,4,6 and 24 h after FAA and daily during IL-2 infusions. Coagulation was assessed by measuring platelets, prothrombin time, par-

Correspondence: G.J.S. Rustin.
Received 10 August 1992; and in revised form 26 January 1993.

tial thromboplastin time, fibrinogen levels and fibrin degradation products. Plasma for estimation of nitrate levels and serum for cytokine levels were stored at − 20° until analysis.

The vascular effect of treatment was assessed indirectly by estimating the extent of tumour necrosis after therapy. Sequential biopsies of cutaneous or subcutaneous tumour nodules were obtained when possible using 2% lignocaine for local anaesthesia. Biopsies were fixed in formalin and subsequently haematoxylin and eosin stained sections were examined. The degree of tumour necrosis was assessed by M.B. and was expressed as a percentage of the total tumour visible.

### *Response and toxicity evaluation*

Response and toxicity were assessed according to standard WHO criteria. A complete response (CR) was recorded if there was disappearance of all known disease for a period of at least 4 weeks and a partial response was recorded if there was a reduction of at least 50% in the sum of the products of the perpendicular diameters of measurable lesions lasting at least four weeks, with no new lesions noted. Hypotension was graded on a four point scale. Grade 1 hypotension was a change of >20 mmHg in systolic pressure; grade 2 hypotension was a change of >30 mmHg, not requiring fluid therapy; grade 3 hypotension was a change in systolic pressure requiring fluid therapy and grade 4 hypotension was a change in systolic blood pressure requiring pressor treatment.

### Results

### *Response*

One patient was withdrawn from the study after day 1 FAA because brain metastases, previously undetected, were observed on CT scan and it was decided that he should receive cranial irradiation. One patient withdrew himself from the study before completing the first cycle of treatment. Of the remaining 21 patients assessable for response, one complete response and two partial responses were noted, giving an overall response rate of 14%. The complete response was in a patient with subcutaneous metastases and liver metastases and has been maintained for 20 + months. One other patient had a complete response in liver metastases (Figure 1) and resolution of >90% of subcutaneous metastases, maintained for 17 + months. The third responding patient had a partial response in cutaneous and nodal disease lasting 15 months. All responses were showing clinical evidence of response to treatment before starting the second course of treatment and the response rate for patients who had no evidence of progression by the start of the second cycle was 3/10 (30%).

### *Toxicity*

A total of 83 courses of FAA (alone or in combination with IL-2) and 39 courses of IL-2 were given. No serious side effects were observed after the day 1 FAA although the majority of patients noted some transient visual disturbance, with flashing lights, during the FAA infusion and 10/23 patients had mild (WHO grade 1/2) gastrointestinal toxicity, which also was shortlived. All patients had at least grade 2 pyrexia during the IL-2 unfusion accompanied, in almost all cases, by lethargy. and myalgia.

However, the most serious side effect observed was a profound drop is systolic blood pressure and the data for hypotension during the first course of treatment are summarised in Table II. No significant drop in systolic blood pressure was noted in any patient after day 1 FAA alone. Significant (grade 3 or 4) hypotension was noted during the day 8–12 infusion of Il-2 in 5/22 (23%) of patients but this resolved quickly with colloid infusion after stopping IL-2. The most profound hypotension was noted 5–24 h after starting the day 15 treatment, which also consisted of FAA followed by an infusion of IL-2. Severe hypotension, requir-

**Figure 1** **a**, Pretreatment scan (October 1990). **b**, Liver metastasis in partial remission (March 1991). Repeat CT scan and ultrasound of liver in October 1991 showed no evidence of liver metastasis.

**Table II** The relationship between treatment and hypotension

| *Treatment* | *No.* | *Grade 3/4 hypotension (%)* |
|---|---|---|
| FAA day 1 | 23 | 0 (0%) |
| FAA day 8 + IL-2 infusion | 22 | 5 (23%) |
| FAA day 15 + Il-2 infusion | 8 | 4 (50%) |
| FAA day 15 alone | 7 | 4 (57%) |

ing pressor support for >24 h, was observed in three of the first eight patients treated. This hypotension was attributed to the reintroduction of IL-2 two days after stopping the initial infusion and, accordingly, the treatment protocol was modified so that the next two patients received no treatment after day 12. As neither patient developed hypotension and as FAA alone given on day 1 was not associated with a significant fall in blood pressure in any patient, day 15 FAA without the IL-2 infusion was reintroduced for the next 7 patients. However, severe hypotension was again noted in 4/7 patients (57%) despite the omission of IL-2. All patients receiving FAA alone 2–4 days after IL-2, experienced fevers and rigors which were similar to those associated with IL-2.

### *Coagulation studies*

Platelet count, prothrombin time, partial thromboplastin time, fibrinogen levels and fibrin degradation products were measured before each FAA treatment, at 1, 2, 4, 6 and 24 h after FAA, and daily during IL-2 infusions for the first ten patients. Apart from a transient thrombocytopaenia in one patient at the end of a five day infusion of IL-2, which resolved quickly spontaneously, no alterations in any coagulation parameters were observed. Coagulation studies were therefore omitted for the subsequent 13 patients.

### *Serial tumour biopsies*

Biopsies of subcutaneous or cutaneous metastatic lesions were obtained at some point during the first course of treatment from a total of ten patients, including the three responding patients, although serial biopsies both before and during therapy were obtained in only five patients (Table III). Of note, necrotic tumour occupying >30% of the specimen was noted in two of the five pretreatment biopsies. Biopsies after day 1 FAA (ie FAA given as a single agent, without IL-2) were obtained at time points ranging from 2 to 52 h after treatment, and one patient had 3 biopsies performed during the 36 h period. Only one specimen, from a patient who had not had a pre-treatment biopsy, had >30% necrotic tumour and, in one patient, the proportion of necrotic tumour was 30–50% in the pretreatment specimen, but <5% in the biopsy obtained 20 h after IL-2. There was no relationship between the degree of tumour necrosis observed in the biopsy specimens and the eventual response to treatment.

### Discussion

Extensive haemorrhagic necrosis is seen in most murine tumours within a few hours of an adequate dose of FAA. This is the first study to investigate whether similar necrosis is seen in human tumours. If it was, any lack of associated tumour response could be due either to the degree of necrosis being inadequate, or the requirement of additional possibly immune factors. There was no evidence of an increase in haemorrhagic necrosis in any of the 20 biopsies taken from 2 to 178 h after FAA. The lack of increased necrosis seen in the biopsies from the three patients whose tumours later responded, suggest that the response was due to a delayed effect, possibly entirely due to the IL-2. However, these results must be interpreted with caution as patchy necrosis can be missed on random samples.

We found no evidence that FAA either given alone or in combination with IL-2 disrupted clotting mechanisms. This information plus the lack of any increase in tumour necrosis in any of our biopsies, indicates that in contrast to its actions in mice, FAA lacks activity against human tumour vasculature. We did not investigate the fibrinolytic pathway which has been shown previously to be deranged by IL-2.

Tumour necrosis factor (TNF) is thought to mediate the haemorrhagic necrosis and anti tumour effect of FAA as this effect is blocked by antibodies to TNF (Mahadevan *et al.*, 1990; Pratesi *et al.*, 1990). We have shown a modest elevation of TNF levels after the combination of FAA and IL-2 but not after IL-2 alone (Haworth *et al.*, 1993). The lack of haemorrhagic necrosis seen in five biopsies taken after combined FAA and IL-2 therapy, could be due to lack of tumour selectivity of any cytokine release.

The major toxicity noted in this study was profound hypotension. This occurred predominantly after the day 15 FAA, irrespective of whether this FAA was followed by an infusion of IL-2 or not. As FAA given alone before the IL-2 infusion did not cause significant hypotension in any patients, the drop in blood pressure observed in the majority of patients after the day 15 FAA must be attributed to a synergistic action between FAA and the IL-2 infusion which had stopped 2 days previously. This affect coincides with the rebound lymphocytosis seen after stopping IL-2. The symptoms are suggestive of a massive cytokine release through the action of FAA on these IL-2 primed cells. Some evidence to support this hypothesis has come from the cytokine estimations performed as part of this protocol (Haworth *et al.*, 1993), where induction of IL-6 and GMCSF appeared to be induced by the day 15 FAA. Nitrate levels were also highest at this time (Thomsen *et al.*, 1992). There was no relationship between the degree of hypotension and the eventual response to treatment.

Because of the impressive activity of FAA against murine tumours, analogues have been produced. It will be essential to look for the occurrence of specific tumour haemorrhagic necrosis and non specific cytokine release when studying these agents in man. As it is difficult to obtain human

**Table III** Necrosis in tumour nodules after FAA and IL-2

| | *Pre* | *FAA* *I* | *FAA* *I* | *IL-2* *IIIII* | *FAA* *I* |
|---|---|---|---|---|---|
| *Responders* | | | | | |
| 1 | <5% | (+2)<5% | (+24) | <5% | |
| 2 | 30–50% | (+20)<5% | | | |
| 3 | <10% | (+52)<10% | | | |
| *Nonresponders* | | | | | |
| 4 | >50% | (+27)<5% | (+29) | <5% | |
| 5 | <5% | (+24)<5% | | | |
| 6 | | (+24) | | | |
| 7 | | (+24)30–50% | | | |
| 8 | | (+5,22 | | | |
| | | and 36)<10% | (+178) | <5% | |
| 9 | | | | | (+24)<5% |
| 10 | | | | | (+24)<5% |
| (hours after treatment to biopsy) | | | | | |

tumour biopsies after therapy, indirect indications of haemorrhagic necrosis such as cytokine, or nitric oxide induction (Thomsen *et al.*, 1992) should be measured.

Objective response to treatment was noted in 3/21 (14%) assessable patients, six of whom were of poor performance status and would have been excluded from many IL-2 studies. This response rate is similar to that reported by Thatcher and colleagues (1990) and not higher than that which would be expected using IL-2 alone. However, the responses were obtained using a much lower dose of IL-2 than that used in the Thatcher's study ($90 \times 10^6$ iu/m$^2$/course compared with $330 \times 10^6$/m$^2$/course), with attendant implications for the cost of treatment. The fact that two patients experienced complete remission of liver metastases which have been maintained for 17 + and 20 + months after just 7 weeks treatment suggests that further studies are required of flavonoids and IL-2.

We are grateful to Nest Howells for supporting the patients treated at Mount Vernon. We thank Lipha pharmaceuticals and Eurocetus for their support. The Cancer Research Campaign supported G. Rustin and S. O'Reilly.

**References**

BIBBY, M.C., DOUBLE, J.A., PHILLIPS, R.M. & LOADMAN, P.M. (1987). Factors involved in the anticancer activity of the investigational agents LM985 (flavone acetic acid ester) and LM975 (flavone acetic acid). *Br. J. Cancer*, **55**, 159-163.

BIBBY, M.C., PHILLIPS, R.M., DOUBLE, J.A., PRATESI, G. (1991). Anti-tumour activity of flavone acetic acid (NSC 347512) in mice – influence of immune status. *Br. J. Cancer*, **63**, 57-62.

CORBETT, T.H., BISSERY, M.-C., WOZNIAK, A., PLOWMAN, J., POLIN, L., TAPAOGLOU, E., DIECKMAN, J. & VLAERIOTE, F. (1986). Activity of flavone acetic acid (NSC 347512) against solid tumours of mice. *Invest. New Drugs*, **4**, 207-220.

DENEKAMP, J. & HILL, S. (1991). Angiogenic attack as a therapeutic strategy for cancer. *Radiother. & Oncol. Suppl.*, **20**, 103-112.

HAWORTH, C., O'REILLY, S.M., CHU, E., RUSTIN, G.J.S. & FELDMAN, M. (1993). Flavone acetic acid with recombinant Interleukin-2 (rIL-2) in advanced malignant melanoma III: induction of high levels of TNF, IL-6 and GM-CSF which coincide with toxicity. *Br. J. Cancer*, **67**, 1346-1350.

KERR, D.J., MAUGHAN, T., NEWLANDS, E.S., RUSTIN, G., BLEEHEN, N.M., LEWIS, C. & KAYE, S.B. (1989). Phase II trials of flavone acetic acid in advanced malignant melanoma and colorectal carcinoma. *Br. J. Cancer*, **60**, 104-106.

MAHADEVAN, V., MALIK, S.T.A., MEAGER, A., FIERS, W., LEWIS, G.P. & HART, I.R. (1990). Role of tumour necrosis factor in flavone acetic acid induced tumour vascular shutdown. *Cancer Res.*, **50**, 5537-5542.

PRATESI, G., RODOLFO, M., ROVETTA, G. & PARMIANI, G. (1990). Role of T cells and tumour necrosis factor in antitumour activity and toxicity of flavone acetic acid. *Eur. J. Cancer*, **26**, 1079-1083.

ROSENBURG, S.A. (1992). The immunotherapy and gene therapy of cancer. *J. Clin. Oncol.*, **10**, 180-199.

SMITH, G.P., CALVELY, S.B., SMITH, M.J. & BAGULEY, B.C. (1987). Flavone acetic acid (NSC 347512) induces haemorrhagic necrosis of mouse colon 26 and 38 tumours. *Eur. J. Cancer Clin Oncol.*, **23**, 1209-1211.

STRATFORD, M.R.L., RUSTIN, G.J.S., DENNIS, M.F., WATFA, R.R., HOWELLS, N. & O'REILLY, S.M. (1993). Flavone acetic acid with recombinant Interleukin-2 (rIL-2) in advanced malignant melanoma IV: Pharmacokinetics and toxicity of flavone acetic acid and its metabolites. *Br. J. Cancer*, **67**, 1351-1355.

THATCHER, N., DAZZI, H., MELLOR, M., GHOSH, A., CARRINGTON, B., JOHNSON, R.J., LORIAUX, E.M. & CRAIG, R.P. (1990). Recombinant interleukin-2 (rIL-2) with flavone acetic acid (FAA) in advanced malignant melanoma: a phase II study. *Br. J. Cancer*, **61**, 618-621.

THOMSEN, L.L., BAGULEY, B.C., RUSTIN, G.J.S. & O'REILLY, S.M. (1992). Flavone acetic acid with recombinant Interleukin-2 (rIL-2) in advanced malignant melanoma II: induction of nitric oxide production. *Br. J. Cancer*, **66**, 723-727.

WILTROUT, R.H., BOYD, M.R., BACK, T.C., SALUP, R.R., ARTHUR, J.A. & HORNUNG, R.L. (1988). Flavone-8-acetic acid augments systemic natural killer cell activity and synergizes with IL-2 for treatment of murine renal cancer. *J. Immunol.*, **140**, 3261-3265.